# Mother Eddy Is Coming To!

**Published:** 1901-03

**Authors:** 


### MOTHER EDDY IS COMING TO!

ONCE more Mother Eddy has come out of her retirement long enough to speak and she has spoken. She has said words of wisdom and has shown more of a spirit of wisdom than is contained in all the closely written pages of the books which bear her name as author. Truly a change—a marvellous change—has come over the spirit of the dreams of this wonderful and mysterious veiled prophetess, whose fire is seen only by the chosen, and whose written words are copyrighted and sold at so much per copy to thousands of followers of the sweet superstition which fosters the crime of law-breaking.

Health Commissioner Wende, recently in Buffalo, made a bitter fight to compel the followers of Eddyism to report contagious diseases. They employed counsel and fought back. They contended that because there was no disease they could not report what did not exist. Several very estimable ladies who hold diploma-mill licenses to practise christian science, made exhibitions of themselves by flaunting their beliefs in the faces of the city authorities and talking very bitterly and with unbridled tongues concerning Dr. Wende's efforts to maintain a low rate of mortality. They denied disease as Peter denied Christ—with open eyes; and they have notoriously been anti-vaccinationists.

How now must they feel to receive this latest proclamation from the fountain head of their beliefs, the Holy of Holies of Eddyism—that mysterious castle in the east, builded in truly regal style with the money wrung from the thousands of deluded creatures who thrive on superstition and exist on beliefs. Remember that christian science has always opposed vaccination; remember that the fundamental principle of christian science has always been its "belief" that disease does not exist; that there being no such condition as disease, contagion could not be reported. Remember this and then read these words of Mother Eddy sent broadcast throughout the country over the wires of the Associated Press.

I have always believed that christian scientists should be law abiding. Rather than quarrel over vaccination, I recommend that if the law demand an individual to submit to this process he obey the law and then appeal to the gospel to save him from any bad effects. This statement should be so interpreted so as to apply, on the basis of christian science to the reporting of contagion to the proper authorities when the law so requires.

This statement is given just as it came from the heart of Mother Eddy, and, *en passant*, it arouses interest in the oft-repeated statement that the woman who is now known as Mary Baker Eddy is not the writer of these beautifully turned sentences which appear in the books and writings bearing her name. Either that or her editor did not have a chance to correct the grammar in her latest pronunciamento.

But, leaving aside these trivial questions of identity, is it not a queer position christian scientists find themselves in just now? They have fought bitterly, as being antagonistic to their beliefs, against doing the very things which they are virtually commanded to do by their demi-goddess up in Concord, New Hampshire.

Mother Eddy is apparently rapidly nearing the mourners' bench. Awhile ago it was reported that she had had her teeth fixed, and had uttered a distinctly human and feminine "ouch" when the dentist's instrument got in its deadly work. Now, she recommends vaccination and the reporting of contagious diseases. Can it really be that the recognised head center of the greatest superstition of centuries is coming to, or is she really and truly alarmed at the prevalence of smallpox and wants to get vaccinated? Who knows? Women are curious creatures, and they are feminine even if they are christian scientists and can see their children die with impassive faces while their thoughts turn to the edited words of a mysterious old woman up in New England for comfort.

---

A REPORT from London states that the county council has virtually adopted a proposal to spend £1,500,000 on a scheme for the better housing of the poor of that great city. This is a matter in which King Edward is greatly interested. It would become the City of New York, and even some other American cities, to take pattern after London in this important sociological movement.

---

THERE are a number of bills in the legislature this winter that need careful watching by the medical profession. The bill to define the practice of medicine is one; a bill introduced by Senator Davis legalising osteopathy is another; one to compel the indorsement of the licenses of other states, no matter what their standards, is a third. The first named should pass; the others should be defeated. The osteopathy bill is absolutely without excuse and is offensive in verbiage and meaning. Senator Davis, its sponsor, has taken a number of steps backward in presenting this measure.

---

THE REED & CARNRICK clinical laboratory, located at 42-46 Germania Avenue, Jersey City, N.J., is doing a splendid work for physicians, in helping them more accurately to diagnosticate disease. The value of such assistance is so far-reaching that it cannot be estimated with readiness. It applies to all diseases of the kidneys with special force, in which the correct methods of the laboratory can only determine the variations in metabolism going on in the body, the relation of nutrition and waste, together with many other important data.

Again, the examination of the blood is constantly increasing in importance, and seems to tell a tale of unerring accuracy in the anemias, leukemias and malarias—one only to be learned through this means.

The importance of sputum examinations in pneumonic affections, too, is essential to correct diagnosis, especially in the early stages of phthisis, or even when it is suspected, for then is the important period to patients and friends.

At the laboratory referred to the prices are so reasonable that the examinations are brought within the money reach of almost everybody. We append a partial schedule of fees for the information of our readers.

*Urine.* —Complete analysis and microscopical examination, including complete qualitative analysis, quntitative analysis of urea, quantitative analysis of sugar or albumin, if present, microscopical examination, Ehrlich's diazo-reaction and other tests, $2.00; quantitative analysis of the following constituents: Uric acid, chlorides, sulphates, phosphates, oxalates, acetone, each, $2.00; bacteriological analysis for tubercle bacillus, $2.00; bacteriological analysis for gonococci, $2.00.

*Blood.*—Number of red and white corpuscles, $2.00; hemoglobin estimation, $1.00; Widal's typhoid test, $2.00; staining and pathological report, plasmodium malariæ, etc., $2.00.

*Serum Culture.*—For bacillus diphtheria, $2.00.

*Sputum.*—Thorough microscopical examination and examination for tubercle bacillus, pneumococus, etc., $2.00.

---

The Maltine Company recently has taken a stand that is calculated to give it a still stronger hold upon the confidence of physicians. It is announced that the company in future will not only refuse to supply the maltine preparations to department stores, but will refuse to supply them to dealers who are known to sell them to department stores. Heretofore, it has been the practice of the company to refer all inquiries, requests for literature or samples from laymen, to local physicians for their information. These and many other methods adopted by this house indicate a desire to conduct its affairs in such a manner as to merit the approbation, not only of the medical profession in general, but of the most pronounced sticklers for ethical regularity between physician and manufacturing pharmacist. We deem this matter of sufficient importance to invite attention to it in these columns.

---

THERE is a judge in Philadelphia by name Wiltbank, who in a recent ruling set himself up as a most heartless person. In the trial of a recent case, a physician who had been called as a witness was late. He had been detained at the bedside of a patient who was dying and explained fully to Judge Wiltbank the reason for his tardy appearance.

Everyone accepted the excuse, except the judge, who promptly fined the physician for contempt of court. Later he remitted the fine and at the same time placed himself in a position, which to say the least is most unenviable, when he said that "it was better that the patient had died rather than that the court should be treated with contempt."

Such a court as exemplified by Judge Wiltbank must need be chary of its contempt, for by his brutal and heartless ruling Judge Wiltbank has placed himself beneath the contempt of every decent-minded human being. He occupies an unique position of loneliness. He is a class unto himself.

